# Supplementary material for: Learning to detect AI texts and learning the limits
Source: PLoS One. 2025 Oct 15;20(10):e0333007. doi: 10.1371/journal.pone.0333007 (PMC12527182; doi:10.1371/journal.pone.0333007)
Supplement: S2 File — All data and scripts used in the study, some additional descriptive statistics, scalable formate figures. (ZIP) [file pone.0333007.s002.tgz › data and scripts/OSF/scripts/analysis/Demographics/correctness_Demographics-age.pdf]

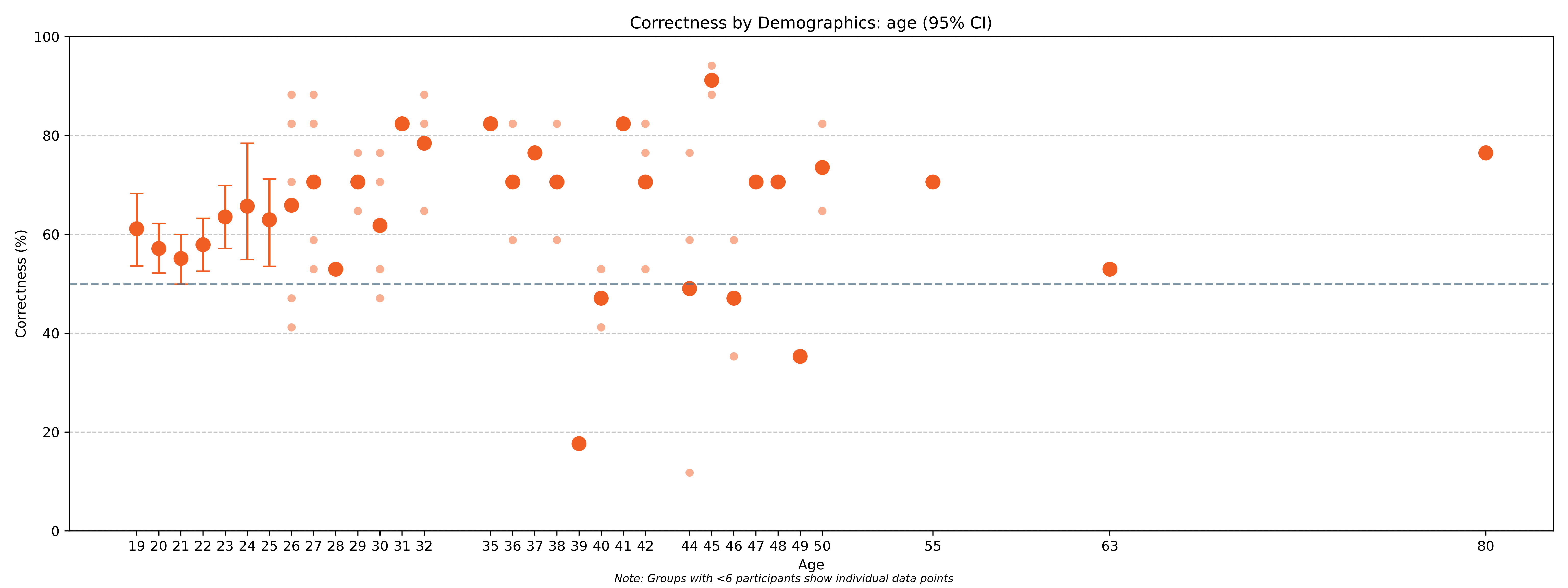

Correctness by Demographics: age (95% CI)
Correctness (%)
0
20
40
60
80
100
19 20 21 22 23 24 25 26 27 28 29 30 31 32 35 36 37 38 39 40 41 42 44 45 46 47 48 49 50 55 63 80
Age
Note: Groups with <6 participants show individual data points
